# Single-particle tracking discloses binding-mediated rocking diffusion of rod-shaped biological particles on lipid membranes

**DOI:** 10.1039/c8sc04033h

**Published:** 2018-11-12

**Authors:** Zhongju Ye, Hua Liu, Fuyan Wang, Xin Wang, Lin Wei, Lehui Xiao

**Affiliations:** a State Key Laboratory of Medicinal Chemical Biology , Tianjin Key Laboratory of Biosensing and Molecular Recognition , College of Chemistry , Nankai University , Tianjin , 300071 , China . Email: lehuixiao@nankai.edu.cn ; https://www.xiaolhlab.cn; b Key Laboratory of Chemical Biology & Traditional Chinese Medicine Research , Key Laboratory of Phytochemical R&D of Hunan Province , College of Chemistry and Chemical Engineering , Hunan Normal University , Changsha , 410082 , China

## Abstract

It has been demonstrated that rod-shaped particles can achieve a high translocation efficiency for gene and drug delivery in biological samples. Previous theoretical calculations also confirmed that rod-shaped particles display higher diffusivity than their spherical counterparts in biological porous media. Understanding the diffusion dynamics of biological and non-biological rod-shaped particles in biological solutions as well as close to the lipid membrane is therefore fundamentally significant for the rational design of efficient cargos. With dark-field optical microscopy, the translational and three-dimensional (3D) orientational diffusion dynamics of individual rod-shaped particles (*i.e.*, *E. coli* and upconversion microrods, UCMRs) in phosphate buffered saline (PBS) and on the lipid membrane are tracked at the single-particle level. In the buffer solution, faster rotation of *E. coli* in the *z* direction was observed even though its dynamics in the *x*–*y* plane is comparable with that of UCMRs. Interestingly, on the lipid membrane, distinct from the confined motion of UCMRs, anomalous rocking diffusion was observed, which might facilitate the subsequent survey of stronger association sites on the two-dimensional (2D) surface. These results would afford deep insight into the better understanding of the translocation mechanism by using rod-shaped particles as a delivery cargo in biological samples.

## Abstract 2

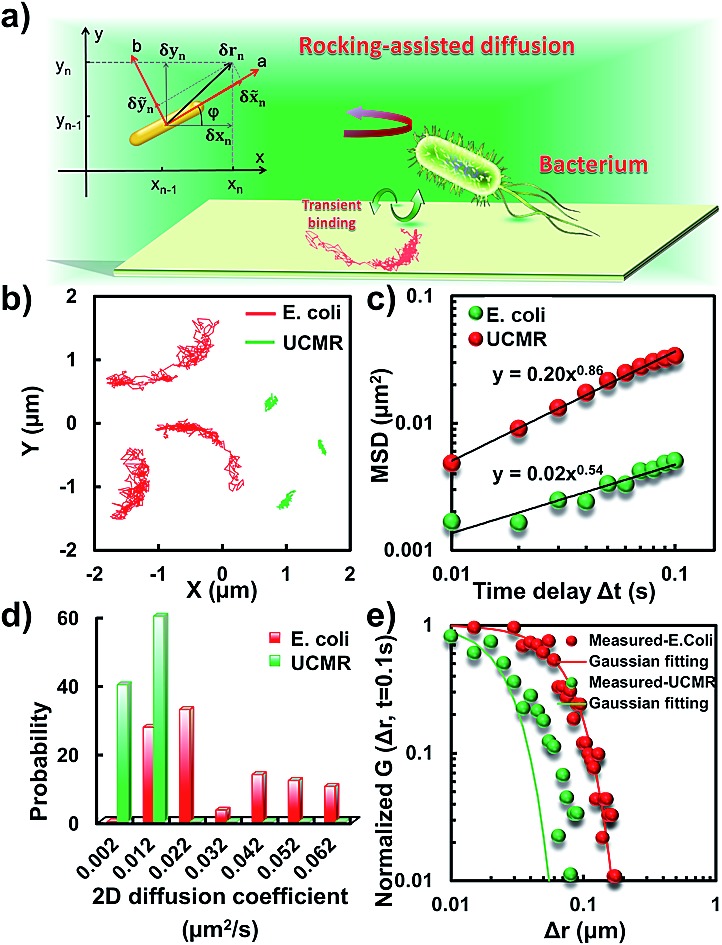
Interestingly, on the lipid membrane, *E.coli* displays anomalous rocking diffusion, which might facilitate the subsequent survey of stronger association sites on the two-dimensional (2D) surface.

## Introduction

The efficient delivery of therapeutic compounds to target tissues or cells is the most significant step for disease treatment.^1^–^3^ Interestingly, several pioneer studies have demonstrated that attenuated bacteria (*e.g.*, *E. coli*) can be adopted as robust delivery vectors for the translocation of functional biomolecules into specific cells with an unprecedented efficiency.^4^–^9^ By taking advantage of the invasive properties of bacteria, Akin *et al.* adopted bacteria to deliver DNA-based model drug molecules (plasmid DNA coding for green fluorescent protein) *in vivo* and *in vitro.*^10^ Analogously, Chen *et al.* found that rod-like inorganic particles with size dimensions comparable to those of bacteria display a good efficiency for gene delivery by using monkey kidney COS-7 cells as the model.^11^

According to previous explorations, the role of the size, morphology and surface chemistry of particles significantly affects the intravenous circulation time, translocation across the cell membrane and intracellular transport routes for drug and gene delivery.^3^,^12^–^15^ Particularly, for rod-like particles, comprehensive theoretical calculations illustrate that the diffusivity is much higher than that of their spherical counterparts in biological matrices.^16^–^18^ Transient adhesive interactions with the host matrix play key roles in such anomalous diffusion.^19^–^21^ Recent explorations also found that elongated rod-shaped inorganic particles exhibit a noticeable cellular translocation efficiency with size dimensions comparable to those of bacteria through carefully manipulating the surface chemistry.^3^ However, different from inorganic particles, biological organisms can swim actively and explore the environment, providing an advantage for gene delivery.^22^–^25^ This is not possible for non-biological vectors. To further extend the potential biological functionality of these rod-shaped particles, it is noteworthy to comprehend the movability of these two structures (biological and non-biological) in solutions and at the biological interface (*e.g.* close to the lipid membrane), where the mechanism of motility controls the primary step for the subsequent cellular uptake process.^26^ However, so far, little knowledge has been gained on the difference in the diffusion dynamics between biological and non-biological particles with similar dimensions particularly close to the 2D interface (*e.g.*, lipid membrane).

On this account, in this work, we explored the translational and rotational dynamics of a bacterium (*i.e.*, *E. coli*) and rod-shaped inorganic particles (with size dimensions comparable to those of the bacterium, UCMRs) in confined (close to the lipid membrane) and free space with dark-field optical microscopy for the first time. According to the single-particle tracking results, it was found that *E. coli* swims and rotates much faster than UCMRs in either free or confined space. In PBS buffer, the rotational motion of *E. coli* in the *z* direction is noticeably more active than that of UCMRs, resulting in a slow sedimentation rate in the biological matrix. However, on the lipid membrane with non-specific adhesive interactions, the bacterium displays anomalous rocking diffusion with occasional tilting motion in the *z* direction. The rotation of flagella on the bacterium surface might be one of the essential sources to promote this kind of motion. For rod-shaped particles, invading into the lipid membrane through anchoring one of the ends to the membrane surface and being followed by continuous rotational motion were reported previously.^21^ In contrast to the bacterium, both of the rotational and translational diffusions of UCMRs were restricted due to the limited thermal energy. These results would afford deep insight into the better understanding of the distinctive translocation efficiency by using these particles as delivery cargos.

## Experimental section

### Chemicals and materials

*E. coli* (wild type shuffle) is grown in culture media (SOS broth, Sigma-Aldrich). NaOH, NH_4_F, NaF, HNO_3_, sodium citrate and ethanol were purchased from Sinopharm Chemical (Shanghai, China). Y(NO_3_)_3_·6H_2_O (99.99%), Er(NO_3_)_3_·6H_2_O (99.99%) and Yb(NO_3_)_3_·6H_2_O (99.99%) were purchased from Aladdin (Shanghai Aladdin Biochemical Technology Co., Ltd.). 1-Palmitoyl-2-oleoyl-*sn-glycero*-3-phosphocholine (POPC) and sulforhodamine 1, 2-dihexadecanoyl-*sn-glycero*-3-phospho-ethanolamine (Texas-Red DHPE, 0.001% wt) were purchased from Avanti Polar Lipids (Avanti Polar Lipids, Inc.). All other chemicals not mentioned here were purchased from Sinopharm Chemical (Shanghai, China).

### Synthesis of NaYF4: 18% Yb: 2% Er rods

The upconversion rods (NaYF_4_: 18% Yb: 2% Er rods) used in this experiment with a diameter of 0.6 μm and length of 2.5 μm were synthesized based on a hydrothermal method that is similar to a procedure described before with minor modifications.^27^ Briefly, an aqueous solution (2.5 mL, 0.2 M) of Y(NO_3_)_3_·6H_2_O, Yb(NO_3_)_3_·6H_2_O and Er(NO_3_)_3_·6H_2_O (lanthanide ion molar ratio, Y/Yb/Er = 80 : 18 : 2) was mixed with 2.5 mL of 2.08 M sodium citrate under stirring for 30 min to form a white solution. Subsequently, 10 mL of 0.625 M NaF solution was injected into the solution. The solution was stirred for another 1 h before the pH of the mixture was adjusted to about 3.0 by adding a dilute HNO_3_ solution. Afterwards, the obtained solution was treated at 180 °C for 6 h. After cooling to room temperature, the products were separated by centrifugation and washed with ethanol three times and then dried at 60 °C in a vacuum. The morphological and size characterization of these UCMRs was performed with a Sirion 200 field emission scanning electron microscope.

### Preparation of the lipid bilayer

The fabrication of the lipid bilayer was based on the self-assembly of small unilamellar vesicles (SUVs) on the surface of a clean glass slide which was described in our previous work.^28^ In brief, POPC was dissolved in a mixture, including chloroform/methanol (1 : 1), for 15 min. Then the solvent was removed by evaporation under vacuum conditions. The lipid film was swollen with PBS buffer and suspended by vortex for 30 min. The SUV solution we used in this experiment was diluted to 0.5 mg mL^–1^ with PBS. A porous polycarbonate membrane (with a pore size of 100 nm) with a mini-extruder apparatus was used to extrude the solution 21 times. The resulting 100 nm SUV solution was stored at 4 °C prior to use.

The lipid bilayer was prepared inside a custom-built flow channel on a clear cover glass slide by self-assembly at 37 °C. 50 μL of the freshly prepared SUV solution was injected into the channel and incubated for 30 min at 37 °C. After that, the channel was washed with PBS. The integrity of the lipid bilayer was measured using Texas-Red DHPE. Texas-Red DHPE was mixed with POPC during the SUV fabrication process. The homogeneously intercalated dye molecules on the lipid bilayer result in an evenly distributed fluorescence signal within the flow channel which demonstrates that the lipid bilayer is successfully generated.

### Single-particle imaging and tracking with a dark-field microscope

Before the single-particle imaging and tracking experiments, the samples were treated as below. The *E. coli* strain was first suspended in culture media with slight shaking at 37 °C for one night. Then, the sample is gently cleaned by centrifugation and re-suspended in the PBS solution at dilute concentrations. 50 mM sodium citrate was used as a protective agent for the as-prepared UCMRs.

The large size and strong scattering properties of these two particles can greatly enhance the signal-to-noise ratio (S/N) of optical imaging. Therefore, good localization accuracy and fast sampling frequency can be achieved. The single-particle tracking experiments were performed on a Nikon Eclipse Ni–U upright optical microscope (Japan). A halogen lamp was focused onto the sample *via* an oil immersion dark-field condenser (NA 1.43–1.20). Scattered light from the sample was collected using a 40× objective, and the successive images were captured with a sCMOS camera (ORCA-Flash 4.0, Hamamastu, Japan) that was mounted on the front port of the microscope within a single frame. The exposure time was set to 10 ms. The pixel size of the sCMOS camera is 6.5 μm × 6.5 μm.

All images were processed with ImageJ (http://rsbweb.nih.gov/ij/). To precisely track the translational and rotational motion of the rods, we chose an ellipsoidal model and adopted the feature point tracking algorithm for the automated detection and quantitative analysis of particle trajectories recorded with the sCMOS camera. The accurate displacement information in the *x*–*y* plane was deduced based on the mass center of the particle. The angle information in the *z* direction can be obtained from the length fluctuation in the long axis.

## Results and discussion

### Transient directed motion of biological particles in the non-adhesive PBS buffer

**Fig. 1:**
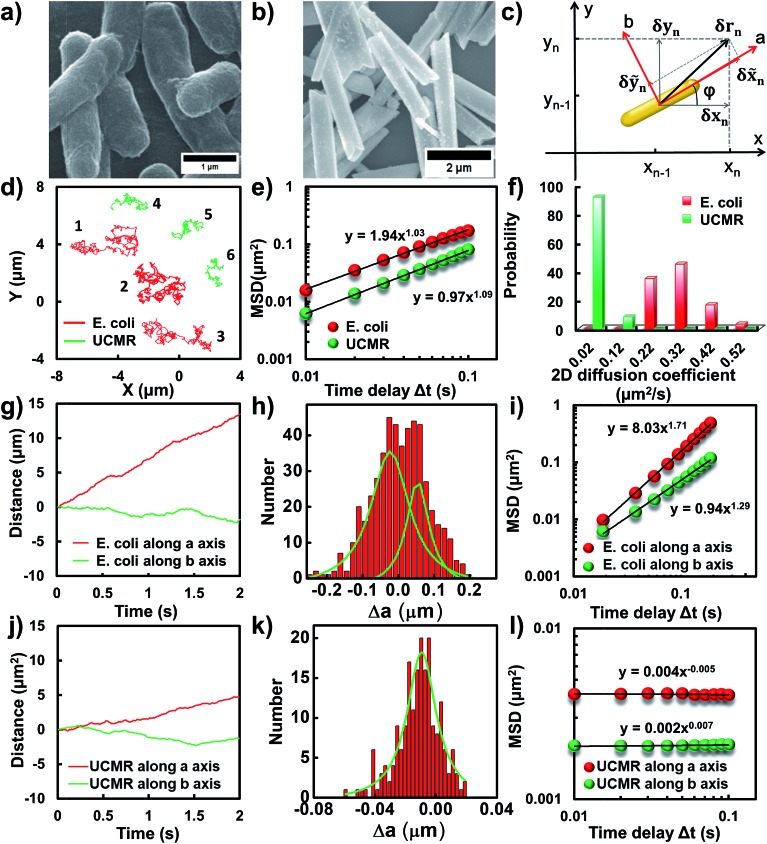
(a and b) SEM images of *E. coli* and UCMRs. (c) The scheme of the Cartesian coordinate system. (d) Representative 2D diffusion trajectories of *E. coli* (red) and UCMRs (green) in the PBS solution. (e) The MSD plots of *E. coli* (red) and UCMRs (green). (f) The 2D diffusion coefficient distributions of *E. coli* and UCMRs. (g and j) The time-dependent diffusion tracks along *a* and *b* axis for *E. coli* and UCMRs, respectively. (h and k) The displacement distributions within a period of 0.01 s along the *a* axis for *E. coli* and UCMRs, respectively. (i and l) The MSD plots along *a* and *b* directions for *E. coli* and UCMRs, respectively.

Flagellar propulsion makes the bacterium (*e.g.*, *E. coli*) very “active” and enables the so-called run-and-tumble movements.^22^ Even though the particles randomly diffuse in free space, at a short time scale, the movement is typically deviated from regular Brownian diffusion. Fig. 1d shows six representative trajectories of *E. coli* (red lines) and UCMRs (green lines) in the PBS solution. Apparently, all of the particles exhibit random diffusion without a noticeable directed or confined behaviour. The movability of *E. coli* is evidently more active than that of UCMRs with a comparable size. This discrepancy can be well rationalized by the fact that the movement of *E. coli* is basically motivated by bioenergy (*i.e.*, propelled by the flagella surrounding the surface). The gradient force or thermal energy has little effect on the movement of the bacterium in contrast to non-biological particles (*e.g.*, UCMRs). Without a chemical gradient, the run-and-tumble movements make *E. coli* swim in various directions, exhibiting some random walks interrupted by occasional directed movements. However, by plotting the mean square displacements (MSDs, swim in various directions, exhibiting some random walks interrupted by occasional directed movements. However, by plotting the mean square displacements (MSDs, 〈ΔΔ*r*(*τ*)^2^〉 = 〈| = 〉 = 〈||*r*(*t* + *τ*) – *r*(*t*)|^2^〉, where Δ, where Δ*r* is the displacement of the particle during the lag time *τ*, *r* is the position of the particle in the *x*–*y* plane, and the brackets manifest an average value over all trajectories.)^29^ from the projected two-dimensional (2D) trajectories of *E. coli* and UCMRs in the *x*–*y* plane, both of these two particles exhibit a well-defined linear correlation between the MSD and lag time ( plane, both of these two particles exhibit a well-defined linear correlation between the MSD and lag time (〈ΔΔ*r*(*τ*)^2^〉 = 4 = 4*D*_*t*_*τ*^*α*^, with an exponent factor *α* of 1.03 and 1.09 for *E. coli* and UCMRs, respectively) (Fig. 1e). The 2D diffusion coefficient (0.49 μm^2^ s^–1^) of *E. coli* is around two-fold higher than that of UCMRs (0.24 μm^2^ s^–1^). Further analyzing the distribution of the 2D diffusion coefficients of these two particles, a much broader distribution with an average value of 0.26 ± 0.07 μm^2^ s^–1^ was observed for *E. coli*, indicative of more active motion (Fig. 1f).

The translocation direction of the particles in the biological matrix is largely affected by the strength of the drag force along the opposite direction. Basically, in homogeneous surroundings, the strength of the drag force for anisotropic nanoparticles in different directions is different. The resulting diffusion direction is the balance between the thermal energy-induced collision from nearby molecules and the ensemble-averaged drag forces in different directions. For example, the drag coefficient of solid ellipsoid particles moving along the lengthwise and sidewise directions is *f*_*a*_ = 4π*ηa*/ln(2*a*/*b* – 1/2) and *f*_*b*_ = 8π*ηa*/ln(2*a*/*b* + 1/2), respectively, where *f* is the drag coefficient, *a* and *b* are the length along the long and short axes of the ellipsoid particle, respectively, and *η* is the viscosity of the solution. According to recent experimental and theoretical results, it has been demonstrated that the diffusivity of particles in non-adhesive porous media would increase monotonically with the aspect ratio of the rods with the same hydrodynamic diameter and decrease with the aspect ratio of the rods with the same minor-axis diameter.^20^ To decipher the details of the discrepancy of the diffusivity of the bacterium and inorganic rods in the PBS solution, we then decomposed the diffusion dynamics of these anisotropic particles into two directions, *i.e.*, along the long axis (a) and short axis (b) as shown in Fig. 1c. Representative decomposed diffusion trajectories along *a* and *b* axes for single *E. coli* and UCMRs are shown in Fig. 1g and j, respectively. Evidently, the diffusion of *E. coli* along the lengthwise direction is higher than that of UCMRs where a comparable step size was noted. On further analyzing the direction-associated diffusion step length along the lengthwise direction (Fig. 1h and k), interestingly, heterogeneous distributions were noted for *E. coli* in contrast to UCMRs, indicative of different diffusion modes shielded by the ensemble MSD analysis. This occasional larger step size along the lengthwise direction greatly promotes the diffusivity of *E. coli* in the medium even though these two particles possess the same aspect ratio around 5–6. Considering the drag force along the lengthwise and sidewise directions, the contribution from this factor is not the key to regulating the diffusion discrepancy. For UCMRs, the diffusivity along *a* and *b* directions is generally in agreement with that in the physical mode in consideration of the viscous drag force (around 1.5 fold larger along the *b* direction).

**Fig. 2:**
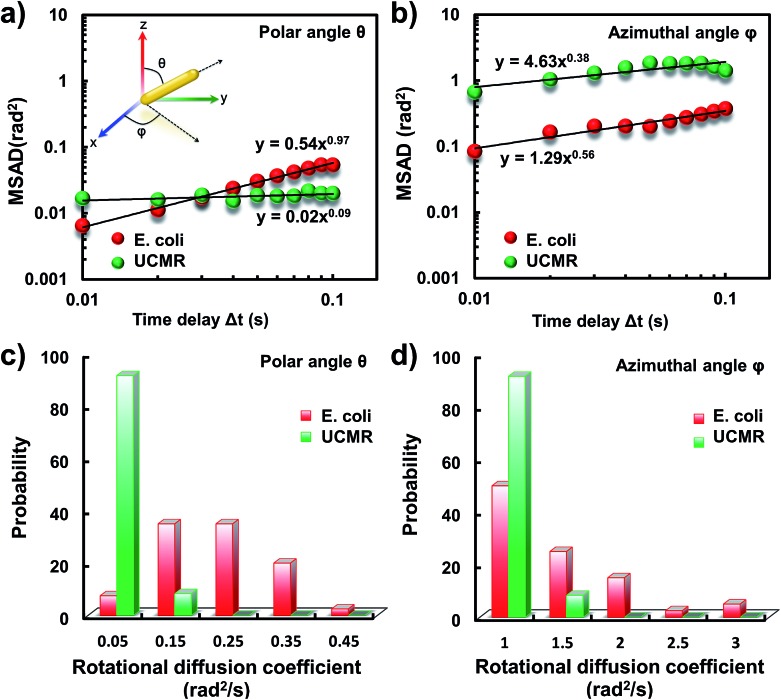
(a) MSAD plots of *E. coli* (red) and UCMRs (green) from the polar angle. (b) MSAD plots of *E. coli* (red) and UCMRs (green) from the azimuthal angle. (c) The rotational diffusion coefficient distributions of *E. coli* and UCMRs from the polar angle. (d) The rotational diffusion coefficient distributions of *E. coli* and UCRMs from the azimuthal angle.

It has been demonstrated that *E. coli* is surrounded by flagella by which it can rotate and propel itself forward. In non-adhesive surroundings, the ensemble-averaged translational diffusion commonly occurs in a random fashion which is in good agreement with the observation as noted above. In addition to the translational diffusion, we then analyzed the 3D rotational dynamics of these two particles as illustrated in Fig. 2. Along the vertical direction (polar angle), the mean square angle displacement (MSAD, . Along the vertical direction (polar angle), the mean square angle displacement (MSAD, 〈ΔΔ*θ*(*τ*)^2^〉 = 〈| = 〉 = 〈||*θ*(*t* + *τ*) – *θ*(*t*)|^2^〉, where , where *θ* is the polar angle of the particle, *τ* is the lag time, and the brackets manifest an average value of the polar angle over all trajectories) of *E. coli* is around one order of magnitude larger than that of UCMRs. Particularly, the exponent *α* is close to 1 ( is close to 1 (〈ΔΔ*θ*(*τ*)^2^〉 = 2 = 2*D*_r_*τ*^*α*^, where *D*_r_ is the rotational diffusion coefficient), representing stochastic angle rotation in the *z* direction. The rotational capability of particles in the polar angle also correlates with the sedimentation rate. For inorganic particles (UCMRs), the rotational motion along this direction is greatly confined (with an exponent factor of 0.09), which might be ascribed to the large net downward force on the particle. In contrast to UCMRs, the rotation of flagella on the bacterium surface assists the suspension of the bacterium in the solution, resulting in active rotational motion in the *z* direction. The distribution of the rotational diffusion coefficient in the polar angle further confirmed this point as shown in Fig. 2c. Obviously, the diffusion coefficient of *E. coli* is larger and broadly distributed than that of UCMRs, manifesting the more active diffusion capability. In the *x*–*y* plane, the motions of these two particles are comparable where only the viscous drag force plays the dominant effect. It is worth noting that the rotational capability in the *x*–*y* plane of *E. coli* is still slightly faster than that of UCMRs (Fig. 2b and d), which might be ascribed to the flagella-assisted active motion. For drug or gene delivery applications, the diffusion capability of the cargo is fundamentally significant, which regulates the circulation time in the biological medium. The active movability of biological particles in translational and rotational modes should therefore play essential roles in promoting delivery applications.

### Anomalous diffusion close to the lipid membrane

**Fig. 3:**
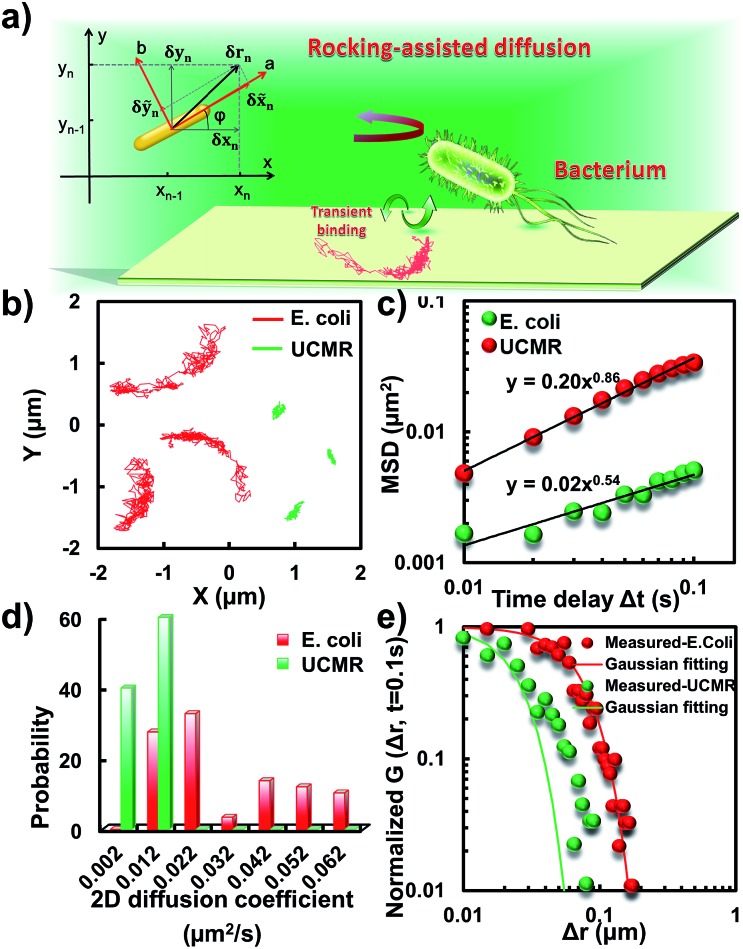
(a) Schematic diagram of the binding-mediated rocking diffusion on the lipid membrane of *E. coli*. (b) The 2D diffusion trajectories of *E. coli* and UCMRs on the lipid membrane. (c) The MSD plots of *E. coli* (red) and UCMRs (green). (d) The distributions of the 2D diffusion coefficients of *E. coli* and UCMRs on the lipid membrane. (e) Double-logarithmic plots of the step size distribution of *E. coli* and UCMRs (normalized by the initial point), respectively.

To translocate target objects into cells efficiently, the key step is to bind and distinguish the penetration site on the 2D lipid membrane. Early explorations by performing a kinetic survey prior to recognizing the final binding site on the infection process of virus toward macrophage cells revealed dynamic association of virus with the cell membrane.^30^,^31^ The active motion of particles such as nanomotors accelerates the translocation process. On account of this, we explored the diffusion kinetics of the particles on artificial lipid membranes by using POPC as the model. Very interesting phenomena were observed where *E. coli* exhibited rocking diffusion at the interface (Fig. 3a and b). In contrast, the majority of UCMRs were confined on the membrane with limited diffusivity. Different from the dynamics in the non-adhesive PBS solution, the 2D translational diffusivity of *E. coli* (0.05 μm^2^ s^–1^) is around one order of magnitude larger than that of UCMRs (0.005 μm^2^ s^–1^). Meanwhile, both of these two particles exhibited confined motion on the lipid membrane with an exponent factor of 0.86 and 0.54, respectively, in the plots of MSD *vs.* lag time ***τ*** (Fig. 3c). By statistically analyzing the diffusion coefficient from them, it is found that UCMRs display a narrow single peak distribution with a mean value of 0.003 ± 0.002 μm^2^ s^–1^ while *E. coli* exhibits a much broader distribution with two noticeable peaks at 0.022 and 0.052 μm^2^ s^–1^, respectively (Fig. 3d). These results illustrate that the active diffusivity of *E. coli* on the lipid membrane is the combination of slow and fast movements. To get deep insight into the details of the dynamics on the lipid membrane, the ensemble-averaged step-size distribution was analyzed which is quantified in terms of the self-part of the van Hove correlation function 
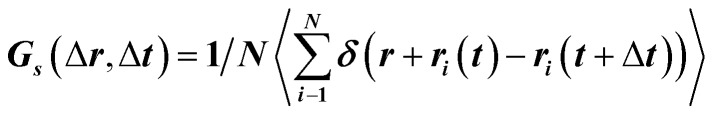
. The estimation determines the probability that a particle has moved a distance of Δ***r*** along the *x* or *y* direction during time Δ***t***. For an apparent Fickian diffusion, the distribution typically follows a Gaussian decay.^32^–^35^ Fig. 3e shows the double-logarithmic plots of the correlation function of UCMRs and *E. coli* with a time duration of 0.1 s. Evidently, the curve decays more slowly than a Gaussian distribution for UCMRs on the lipid membrane. This is contrary to the case of *E. coli*, where the step size is much broader and exhibits a characteristic Gaussian shape, indicative of apparent Fickian diffusion. The non-Gaussian distribution of the UCMRs might be largely attributed to the gravitational drag force close to the interface. Compared with the UCMRs, the flagellar propulsion of *E. coli* balanced parts of the subsidence effect, leading to more active motion at the interface.

**Fig. 4:**
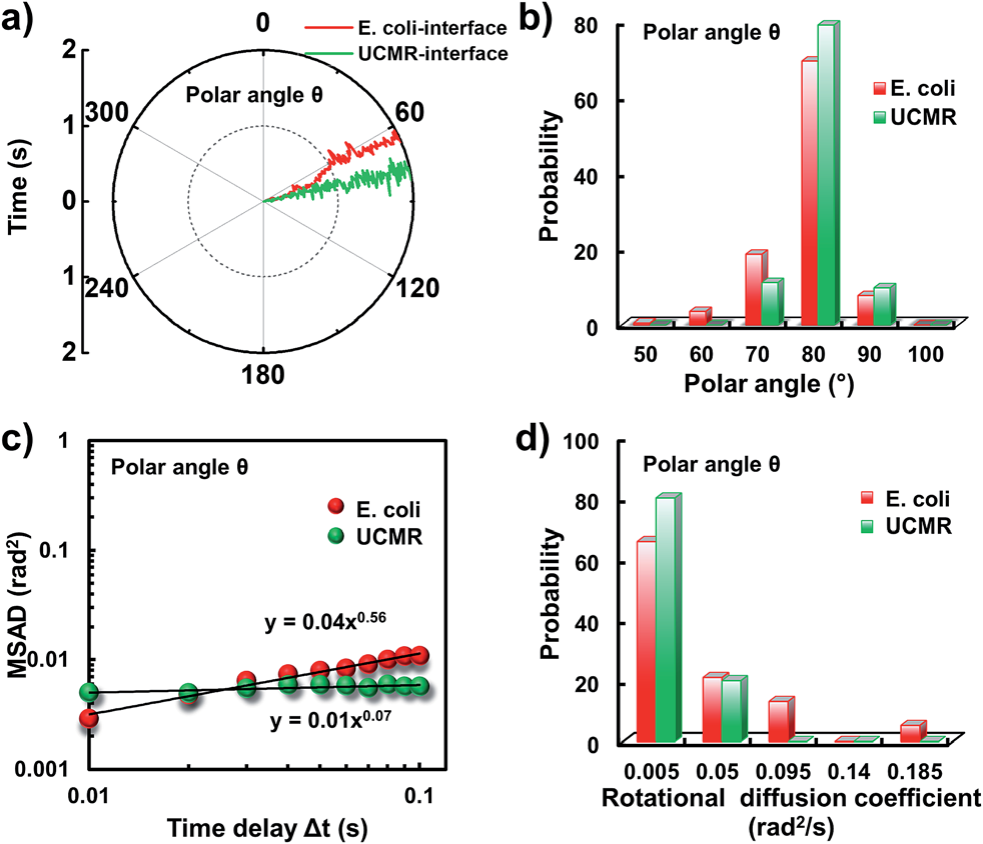
(a) The angular rotational trajectories of *E. coli* and UCMRs on the lipid membrane along the *z* direction. (b) The polar angle distributions of *E. coli* and UCMRs. (c) Representative MSAD plots of *E. coli* and UCMRs from the polar angle on the lipid membrane. (d) The distribution of the rotational diffusion coefficients of *E. coli* and UCMRs on the lipid membrane along the *z* direction.

**Fig. 5:**
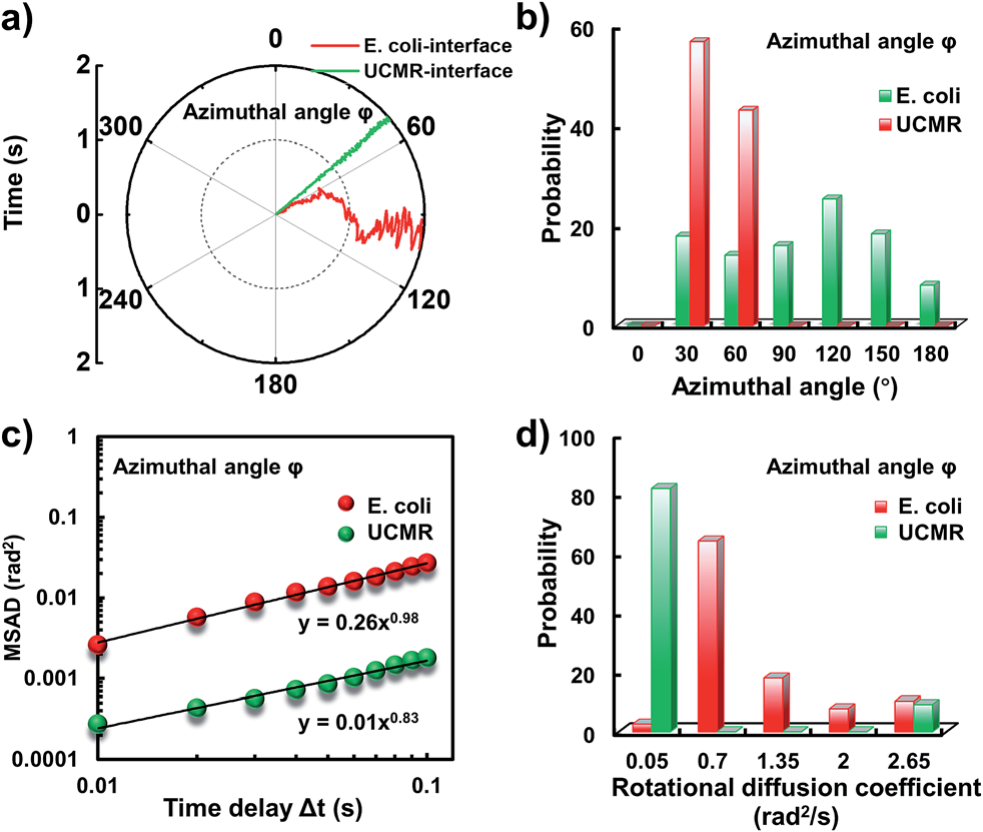
(a) The angular rotational trajectories of *E. coli* and UCMRs on the lipid membrane in the *x*–*y* plane. (b) The azimuthal angle distributions of *E. coli* and UCMRs. (c) Representative MSAD plots of *E. coli* and UCMRs from the azimuthal angle on the lipid membrane. (d) The distribution of the rotational diffusion coefficients of *E. coli* and UCMRs on the lipid membrane in the *x*–*y* plane.

Since the rotational motion might synergistically affect the translational movement, we further determined the rotational dynamics on the lipid membrane. Representative rotational trajectories in the *z* direction (the polar angle) and *x*–*y* plane (the azimuthal angle) are shown in Fig. 4a and 5a, respectively. In the *z* direction, the rotational movements of these particles are confined with an average angle distribution close to 80° (Fig. 4b). In contrast to *E. coli*, the tilting movement of UCMRs is significantly inhibited which might be essentially ascribed to the gravitational force when the particles sediment close to the interface. In the case of *E. coli*, although the movement in the *z* direction is one-order of magnitude decelerated in contrast to that in the free solution, occasional jumps in the *z* direction were observed, which is reflected in the MSAD assay in the *z* direction (Fig. 4c). The angle distribution in this direction also confirms this argument where a larger tilt angle distribution was found (Fig. 4b). The statistically analyzed rotational diffusion coefficient in the polar angle is shown in Fig. 4d. Evidently, a long tail with faster rotational movements was observed for *E. coli*.

Interestingly, in the *x*–*y* plane, the rotational movement of *E. coli* is much faster than that of UCMRs (one-order of magnitude larger) with the exponent factor in the MSAD plot close to 1 (Fig. 5). This active motion might be propelled by the rotational movement of the flagella on the bacterium surface. A much broader angle distribution and faster rotational diffusion were found for *E. coli* in the azimuthal angle (Fig. 5b and d). These observations illustrate that even though the rotational movement of *E. coli* on the lipid membrane is confined, the motion of the flagella might greatly assist the active motion close to the interface. However, it is still interesting why most of the *E. coli* exhibit circular rocking diffusion at the interface as shown in Fig. 3b.

### Transient binding-induced rocking diffusion of *E. coli* on the adhesive interface

**Fig. 6:**
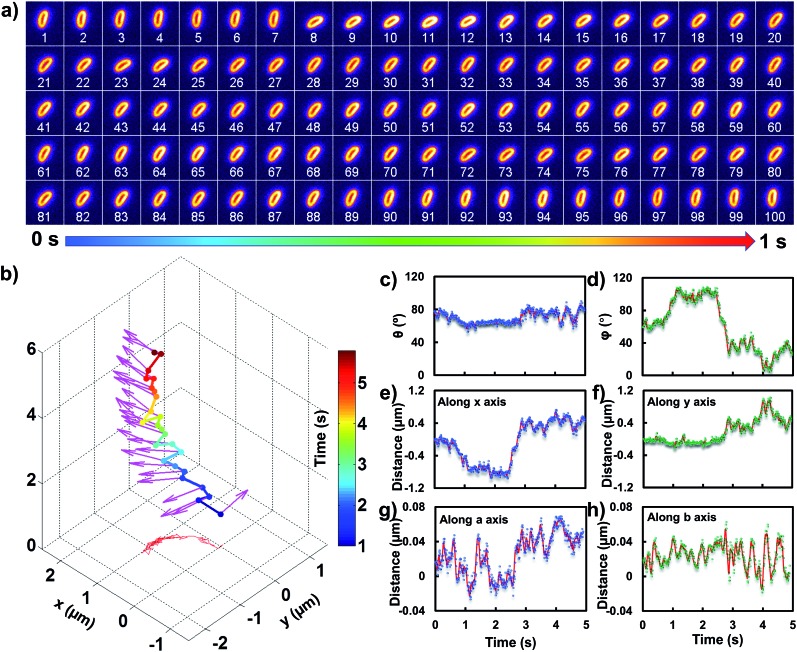
(a) Frame-by-frame images of the rotational and translational diffusion of *E. coli* on the lipid membrane with an observation time window of 1 s. (b) The time-dependent trajectory of *E. coli* on the lipid membrane. The 3D orientations from different time periods are marked with red arrows. (c)–(h) The time-dependent trajectories from the polar angle, the azimuthal angle, the walking displacement along *x* and *y* axes, and the walking displacement along *a* and *b* axes, respectively.

In order to comprehend the mechanism of the anomalous rocking diffusion at the interface, we further inspect the detailed diffusion trajectory of single *E. coli*. Fig. 6a displays the rotational dynamics of *E. coli* with a time resolution of 100 Hz. Evidently, reversible rocking rotation was noted in the *x*–*y* plane. Fig. 6b depicts the detailed time-dependent angular rotation process (denoted by arrows) and translational diffusion trajectory of the bacterium. The orientation in the *x*–*y* plane was continuously changed in association with the occasional tilting motion in the *z* direction. For the convenience of observation, the rotational and translational trajectories were plotted as a function of time and are shown in Fig. 6e–h. Within 1 s, the polar angle didn't change greatly (Fig. 6c). The orientation direction in the *x*–*y* plane was almost the same. The abrupt change in the polar angle is usually associated with fluctuations in the azimuthal angle (Fig. 6d). Particularly, within the time period between 1 and 3 seconds, the tilt angle is relatively large, resulting in faster rotational capability in the *x*–*y* plane. The reversible change in the rotational direction in the *x*–*y* plane is well reflected in the diffusion process in *a* and *b* directions. Within the window of 3–5 s, it is obvious that the bacterium swayed continuously in the *z* direction, indicative of the adsorption and desorption processes at the interface. The tilted polar angle released the rotational degree of freedom in the *x*–*y* plane. Since one of the ends might still associate with the lipid membrane, the diffusion was restricted.

To date, many research studies and models have been exploited to explain the dynamics of these delivery cargos.^36^–^43^ Most bacteria exhibit random walk in homogeneous environments. Bacterial random walk is often biased when the environment changed.^44^*E. coli* is surrounded by flagella by which it can rotate and propel itself forward, such as the well-known run-and-tumble mobility. When running, all the flagella rotate counterclockwise (CCW) and form a bundle. It gives the swimmer a propulsive force which is balanced by the viscous fluid drag force. The bacterium can swim at a steady speed in a straight line until it tumbles. The flagellar bundles disassembled when they encountered some external stimuli, leading to reorientation, and thus they are kept away from unfavourable environmental conditions by changing the swimming direction. When swimming in a non-uniform environment, such as the case close to the lipid membrane, the mobility of the organism may change in a tempo-spatially dependent manner.^39^ Taking into account these considerations, the anomalous behaviour of *E. coli* on the lipid membrane can be well delineated as below. Given one of the terminals of the *E. coli* adhered onto the lipid membrane, the rotational motion of the flagella propels the *E. coli* to move circularly together with slight sliding motion. Provided the flagellar bundles disassembled, occasional tilting jumps in the *z* direction might take place in association with diffusion direction transition, resulting in the anomalous rocking diffusion close to the interface.

Gene or drug delivery with functional materials is a realistic prospect for the treatment of cancers and has being investigated and employed clinically for a wider range of diseases in recent years.^45^,^46^ However, some grand challenges still exist, such as the poor loading efficiency, limited biocompatibility, and fast sedimentation rate in biological fluids. The development of efficient and convenient vectors for delivery purposes is now widely acknowledged.^47^ In general, vectors for delivery applications can be classified into non-biological and biological models. As demonstrated in previous studies, non-biological vectors such as UCMRs are attractive for delivery applications because of their safety profile. However, the efficiency of cellular translocation from current non-biological approaches is comparatively poor in contrast to that of biological vectors such as bacteria, thereby limiting clinical efficacy and restricting the range of applicable therapeutic approaches.^48^ Therefore, a better understanding of translational and rotational dynamics of biological and non-biological vectors in biological matrices should provide great significance on the rational design of efficient delivery cargos.

## Conclusions

In summary, with dark-field optical microscopy, the rotational and translational diffusion dynamics of *E. coli* and UCMRs in the PBS solution and close to the lipid membrane were tracked at the single-particle level. In the bulk surrounding, both of the biologically active and non-biological particles exhibited apparent Brownian diffusion according to the MSD analysis. The rotational dynamics in the *x*–*y* plane of these two particles were comparable. However, along the *z* direction, *E. coli* exhibited more active motion, resulting in a slow sedimentation rate in the bulk solution, which might be helpful for the improvement of the circulation time in biological fluids. On the lipid membrane, anomalous confined motions were observed where most of the *E. coli* displayed transient binding-mediated rocking diffusion at the interface while the majority of UCMRs were confined. This kind of motion might promote the rapid survey and recognition of effective binding sites on the lipid membrane, which is normally associated with the endocytosis process. These observations might shed new light on the translocation mechanism of rod-shaped particles in biological media and provide guidelines for the rational design of efficient delivery cargos.

## Conflicts of interest

There are no conflicts to declare.
